# On the design of clone-based haplotyping

**DOI:** 10.1186/gb-2013-14-9-r100

**Published:** 2013-09-12

**Authors:** Christine Lo, Rui Liu, Jehyuk Lee, Kimberly Robasky, Susan Byrne, Carolina Lucchesi, John Aach, George Church, Vineet Bafna, Kun Zhang

**Affiliations:** 1 Department of Computer Science and Engineering, University of California, San Diego, La Jolla, CA, USA; 2 Department of Bioengineering, University of California, San Diego, La Jolla, CA, USA; 3 Department of Genetics, Department of Genetics, Harvard Medical School, Boston, MA, USA; 4 Wyss Institute for Biologically Inspired Engineering, Harvard University, Cambridge, MA, USA; 5 The Bioinformatics Program, Boston University, Boston, MA, USA; 6 Present address: Expression Analysis, A Quintiles Company, Durham, NC 27713, USA

## Abstract

**Background:**

Haplotypes are important for assessing genealogy and disease susceptibility of individual genomes, but are difficult to obtain with routine sequencing approaches. Experimental haplotype reconstruction based on assembling fragments of individual chromosomes is promising, but with variable yields due to incompletely understood parameter choices.

**Results:**

We parameterize the clone-based haplotyping problem in order to provide theoretical and empirical assessments of the impact of different parameters on haplotype assembly. We confirm the intuition that long clones help link together heterozygous variants and thus improve haplotype length. Furthermore, given the length of the clones, we address how to choose the other parameters, including number of pools, clone coverage and sequencing coverage, so as to maximize haplotype length. We model the problem theoretically and show empirically the benefits of using larger clones with moderate number of pools and sequencing coverage. In particular, using 140 kb BAC clones, we construct haplotypes for a personal genome and assemble haplotypes with N50 values greater than 2.6 Mb. These assembled haplotypes are longer and at least as accurate as haplotypes of existing clone-based strategies, whether *in vivo* or *in vitro*.

**Conclusions:**

Our results provide practical guidelines for the development and design of clone-based methods to achieve long range, high-resolution and accurate haplotypes.

## Background

Current whole genome sequencing (WGS) technologies can provide genotype information of the human genome with very limited haplotype information. Understanding the human genome on the haploid level is important in the development of personalized medicine, as haplotypes can help elucidate genetic variants associated with gene expression, long-range interaction, and susceptibility of humans to disease [1]. Many computational and experimental approaches have been developed to reconstruct phase information for diploid genomes, and each has its strengths and limitations. For example, population-based methods are commonly used to infer the haplotypes based on genotypes of a population, but have limited success in phasing rare or individual-specific variants [2-5]. Sequencing technologies allow us to 'assemble’ haplotypes by chaining together variants that appear on the same read; however, the length of the haplotype is largely dependent on read length and sequence coverage [6], making it challenging to phase with shorter read information. Haplotype contigs assembled from shotgun sequencing reads are typically orders of magnitude shorter than genomic contigs, owing to the sparse distribution of heterozygous variants required for haplotype assembly. Direct resolution of allelic haplotypes can be achieved by physical separation of individual chromosomes using microfluidic devices or microdissection, but amplification bias of the isolated chromosomes leads to low resolution [7-9].

Another widely used approach is clone-based haplotyping, first introduced by Burgtorf *et al*. [10]. The basic principle behind this method involves constructing clone libraries that will extract long subsections of a haploid and pooling together several clones for sequencing. As long as the clones within a pool do not overlap, the clones can be computationally reconstructed from shorter sequencing reads and assembled into longer haploid sequences. Alternative implementations of the clone-based haplotyping method [11-13] mainly differ in how clones are generated (affecting the length of the clones) and the number of pools sequenced. For example, in a study by Suk *et al*., fosmid clones with an average length of 40 kbp were combined into 288 pools, with 5,000 clones per pool [12], and the N50 length of the assembled haplotypes was 1 Mbp. Several conceptually similar haplotyping methods have recently been reported, which fragment genomic DNA *in vitro* and then pool together the fragments for sequencing. From example, the long fragment read (LFR) method was used in one study [13] to generate haploid fragments of length (*L*) 10 to 300 kbp, which were combined into 384 pools with around 5,000 to 10,000 fragments per pool. The resulting N50 length of the assembled haplotypes ranged from 400 kbp to 1 Mbp. Another method generated haploid fragments of average length (*L*) = 13.8 kb, leading to haplotypes with comparable N50 values [14]. Most recently, research using Moleculo technology (Illumina Inc., San Diego, CA, USA) reported fragments with *L* = 6 to 8 kb [15], possibly reaching up to 10 kb.

The differences in the experimental designs of these studies directly affect the cost versus haplotype length trade-off. Previous clone-based haplotyping experiments did not explicitly consider how their parameter choices affect the cost versus haplotype length trade-off, and often used the same design criteria as those used for sequence assembly. Note that there is a major difference between sequence assembly and haplotype assembly; sequence assembly relies on partially overlapping short sequences of typically 20 to 70 bp in length, whereas haplotype assembly depends on multiple adjacent heterozygous variants at a typical spacing of 1.5 kb, which is a much more stringent requirement.

Here, we pursue a parameterized approach to haplotype assembly. We considered the following parameters: clone length (*L*); number of clones per pool (*n*); number of pools (*p*); and sequence read coverage per pool (*r*). The use of parameters allowed us to compare the different methods (Table 1) and understand the effects of haplotype length on different parameters. Please note that we use the term 'clone’ in this paper because we were designing explicitly for clone-based haplotyping; however, a 'clone’ can refer more generally to any type of haploid subsequence of the genome, regardless of how it was obtained (that is, *in vitro* or *in vivo*).

**Table 1:** Comparison of different clone-based haplotyping protocols

	**Kitzman *et al*. **[11]**(fosmid)**	**Suk *et al*. **[12]**(fosmid)**	**Peters *et al*. **[13]**(LFR)**	**Kaper *et al*.**[14]	**Lo *et al*. **[16]**(BAC)**
*n*: Number of clones per pool	5,000	5,000	5,000 to 10,000^a^	16,377^b^	5,000
*L*: Exp(clone length), kbp	37	40	60	13.8	140
*p*: Number of pools	115	288	384	192	24
*c*: Exp(clone coverage) = $\frac{\mathrm{nLp}}{G}$	7.1	19.2	57.6	14.5	6.0
*c*_*p*_: Exp(clone coverage per pool) = $\frac{\mathrm{nL}}{G}$	0.06	0.07	0.15	0.075	0.25
*P*_O_: overlap probability = $1-e^{-c_{p}},\%$	11.31	13.06	25.92	13.93	39.35
Exp(haplotype length), bp	2.05 × 10^7^	4.37 × 10^10^	5.30 × 10^16^	4.89 × 10^9^	3.42 × 10^5^
Simulated haplotype length, bp	825,046	2,486,692	8,585,663	300,336	2,210,343
Actual haplotype length, bp	386,000	959,175	411,000^c^	358,000	2,640,036

*Abbreviations:* BAC, Bacterial artificial chromosome; LFR, LFR, Long fragment reads.

^a^Estimated given that each pool contains 300 to 600 Mbp and *L* = 94,000.

^b^Estimated by dividing 226 Mbp (number of bases covered in a pool) by *L* = 13.8 kbp.

^c^Actual haplotype length for NA20431.

We started with the assumption that once the clone library is prepared, the cost is simply a function of the number of pools (*p*). However, our calculations are useful for understanding other trade-offs as well. Although intuitively, larger clone size (*L*) leads to longer haplotype length, the assembled haplotype length depends upon the combination of parameters *L, n, p*, and *r* in a non-trivial fashion. Connecting overlapping haploid clones generates long haplotypes. As mentioned above, haplotype assembly is unlike sequence assembly, where *L* only needs to be long enough to span the longest repetitive sequence. For haplotype assembly, the clones must be long enough to span adjacent heterozygous variants. Therefore, the first step in a good design is to make *L* as large as possible within the constraints of available technology and cost. Next, to maximize the chance of getting overlapping clones, the total clone coverage ($c=\frac{\mathrm{nLp}}{G}$)should be maximized. At the same time, overlapping clones within a pool may lead to heterozygous calls that are not informative for haplotyping, implying that clone coverage within a pool ($c_{p}=\frac{c}{p}$) should be kept low. The naive way to accomplish these design objectives is to keep *n* low and *p* high, which in turn, increases the cost of the experiment.

We studied the *p* versus length trade-off by simulating clones under different experimental parameters, and assembling haplotypes assuming a known distribution of variants. To experimentally validate the effect of using larger clone size (*L*), we performed an experiment with *p* = 24 pooled bacterial artificial chromosome (BAC) clones from a Caucasian male sample, NA20431, of the Personal Genome Project (designated PGP1). BAC clones are longer (*L* = 100 to 300 kbp [17]) than fosmid-based clones (*L* = 40 kbp) and LFRs (*L* = 60 kbp). To keep sequencing costs low, we used additional reads from single low-pass WGS in addition to modest coverage in each pool. Even with low sequencing cost (*p* and sequence coverage), we identified the assembly of accurate haplotypes that were more than 2.5 times longer than previously reported ones. Our results also suggest important design principles for clone-based haplotype assembly: for long haplotyping*, L* should be as high as possible within the bounds of technology; it is possible to achieve long haplotypes with a much smaller *p* (and hence lower experimental cost) than was implemented in the previous efforts; and, there is a direct trade-off between depth of sequencing per pool (*r*)*,* and the length and resolution of haplotypes (fraction of variants phased), but modest sequencing depth is sufficient.

In addition to length, we also took into account the accuracy of the generated haplotypes. The two types of errors that can arise in haplotyping are mismatches and switches. Mismatches are defined as single nucleotide differences between the assembled haplotype and the true haplotype, and are probably caused by erroneous base calls. Switch errors are defined as positions where a crossover in haplotype orientation is needed to recover the true phase. To test the accuracy of the haplotypes, we need to compare the generated haplotypes with the true haplotypes (that is, haplotypes from trio data). However, the true haplotypes are not always known, and without knowledge of the ground truth, the best we could do is compare with haplotypes obtained via other methods.

## Results

### Design of experiment for clone-based haplotyping

To assemble long and accurate haplotypes, the clone coverage, *c*, (that is, the average number of clones covering each genomic position) must be high enough so that overlapping clones from different pools can be assembled to form longer haplotypes. For a genome of length *G*, the expected clone coverage is given by $c=\frac{\mathrm{nLp}}{G}$. Assuming that clones of fixed length *L* arrive at random and overlapping clones come from different pools, the overlapping clones assemble into longer contigs. The Lander and Waterman estimate [16] for expected length of a contig is given by:

(1) $$\mathrm{Exp}\left(\mathrm{contig}\;\mathrm{length}\right)=\frac{L\left(e^{c}-1\right)}{c}.$$

### Effect of clone length (*L*)

**Figure 1:**
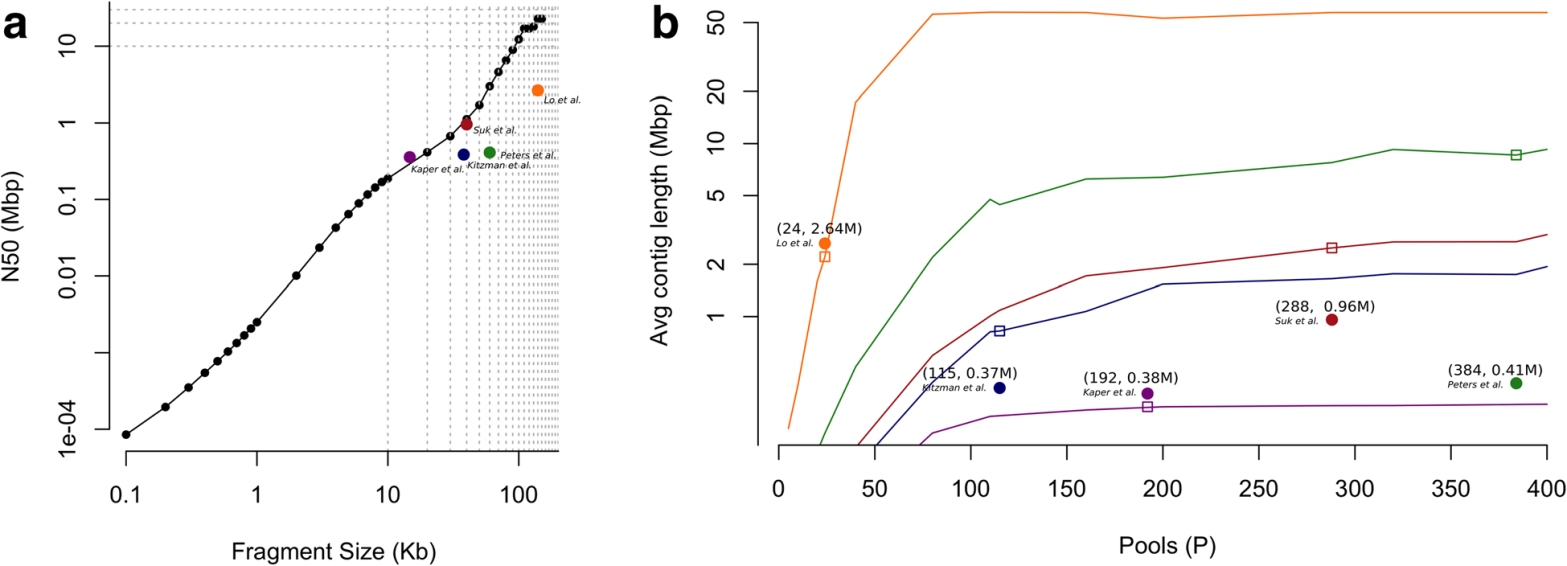
**Expected contig length for various clone-based haplotyping designs. (a)** Log-log plot of the maximum achievable haplotype N50 length for different values of clone length (*L*) (assuming a distribution of heterozygous variants obtained from Complete Genomics Institute (CGI) whole genome sequencing (WGS) on chromosome 1 of sample NA20431 of the Personal Genome Project (designated PGP1). This plot suggests a power law relationship between haplotype N50 length (N50) and clone length (*L*), which is characterized by N50 being approximately *L*^1.42^. Note that achieved haplotype lengths (filled circles) may not reach the maximum length, owing to smaller numbers of pools or low fraction of the variants recovered. **(b)** Simulated haplotype length versus the number of pools (*p*) for given values of *L* (shown in different colors). In all cases, except one (magenta), the number of clones per pool (*n*) is 5,000 (*n* = 16,800 for magenta). The curves reach saturation when all variants that are less than distance *L* apart are connected in a contig. Simulations are performed using the distribution of heterozygous variants obtained from CGI WGS on chromosome 1 of PGP1. The squares represent the simulated estimate given parameter settings of several clone-based haplotyping experiments, while the circles show the reported N50.

The Lander and Waterman estimate allows for a quick comparison of different strategies, and suggests that increasing coverage *c* can compensate for low *L* (see Additional file 1: Figure S1). However, this does not model an important aspect of both sequence and haplotype assembly. In sequence assembly, *L* must be long enough to span repeats in order to permit unambiguous assembly. Once *L* exceeds the length of known repetitive sequence (about 10 kbp for humans in order to span long interspersed nuclear elements (LINEs)), increasing *L* further has diminishing returns [18]. However, in haplotype assembly, overlaps are informative for phasing only when they cover heterozygous variants. If two adjacent heterozygous variants are further apart than the length of a clone, they cannot be linked into a single haplotype. Based on the observed variant distribution in the human genome, no saturation is seen even with very high values of *L* (Figure 1a). Thus, *L* must be chosen as high as is technologically possible. We show the affect of long clone size on haplotype assembly by using available BAC clones (140 kbp), which are longer than clones from previous approaches (Table 1).

### Effect of pool number (*p*)

The clone coverage must be high in order to form long contigs, but overlapping clones within a pool result in heterozygosity, and are not informative for haplotyping. Denoting the coverage per pool as cp=cP, the probability of overlap for a clone is given by:

(2) $$P_{o}=1-e^{-2c_{p}}$$

Previous clone-based methods, [11-13] (Table 1) all kept *c* high and *c*_*p*_ low by keeping *p* high and *nL* low. As each pool must be sequenced independently, the cost increases linearly with p. To keep sequencing costs low, we considered the effect of overlaps within a pool explicitly. As a first approximation, we simply discard clones that overlap within a pool. Thus, the number of clones per pool is reduced to *n*' = *n*(1 - *P*_*O*_), yielding a new coverage of $c'=\frac{n'\mathrm{Lp}}{G}$. Figure 1b shows this '*p* (or cost) versus contig-length’ trade-off, and clearly shows that the previous approaches (denoted by circles) used many more pools than necessary for their specific clone length choices. Here, we worked with a relatively low value of *p* = 24, which kept costs low. We additionally improved haplotype contig lengths by not discarding overlapping clones in a pool, but separating them computationally (see detailed Results below, and Methods).

Another consideration in the design is the recovery of heterozygous variants. For haplotyping, the heterozygous variants of the individual must be linked, and therefore the variant must be sampled from both parental chromosomes. By contrast, the homozygous variants (reference or non-reference) can be filled in subsequently, and it is only necessary to sample the variant on one chromosome. The expected percentage of heterozygous variants that are sampled by clones from both chromosomes is given by:

(3) pv=1-2e-v2

We worked with low values of *c* = 6x, thus we expected only 90% of the heterozygous variants to be recovered. To recover more heterozygous variant locations, we augmented the detected heterozygous variants by using additional WGS data of the same individual.

### Effect of sequence read coverage per pool (*r*)

The final parameter of interest is the read coverage per pool, *r*. Increasing *r* increases the sequencing cost per pool, but low values of *r* can affect clone reconstruction, and thus haplotype length and resolution. For example, low values of *r* decrease the resolution of the clones (that is, not all bases spanned by a clone will be covered by a read) and make it difficult to detect clone boundaries. At the same time, increasing *r* has diminishing returns for increasing cost. In particular, assuming a *Poisson* distribution with parameter *r*, the probability that a position is covered by *k* reads is given by:

(4) pr≈e-rrkk!

For *r* = 6x, Equation 4 suggests that 84% of the base pairs spanned by a clone are covered by four or more sequence reads. However, the actual coverage (see Additional file 1: Figure S2) suggested that the coverage distribution is not Poisson. In fact, only 65% of the base pairs spanned by a clone were covered by four or more sequence reads. The bias in coverage could be attributable to a variety of factors, such as amplification bias and filtering of reads in order to control for repeats. To capture all these factors (including *r*, amplification bias, filtering of reads, and variants), we worked directly with the parameter *f* (the fraction of heterozygous variants recovered in a clone). In our experiments, *r* was 6x and *f* was 65%. We studied the effects of *f* on haplotype length and haplotype-resolution via simulations (see Additional file 1: Figure S3 and Figure S4), and our results showed that modest values of *f* (or sequencing depth *r*) and *p* could be used to achieve long haplotypes with high resolution.

### BAC pool construction

Our analysis suggested that using a limited number of pools of larger clones can lead to longer haplotypes than using many pools of smaller clones or fragments. To provide experimental support for this prediction, we implemented the clone-based haplotyping strategy using a set of BAC clones. We started with existing BAC clones constructed from high molecular weight PGP1 genomic DNA and individually maintained in 384-well plates for other purposes. Given that the mean length of a BAC clone is 140 kbp (*L*), 384 BAC clones in one plate amount to 54 Mbp, approximately 1.7% of the 3.2 Gbp human haploid genome. Additional pooling of fourteen 384-well plates, containing a pool of 5,376 (*n*) BAC clones, would be expected to cover about a quarter of the human genome. With a total of 24 (*p*) pools, the expected clone coverage (*c*) was 6x. The PGP1 BAC library was constructed for multiple purposes and maintained as one clone per well in 384 wells, which involved a high cost (>US$50,000) for handling individual wells. For haplotyping purposes, we estimated that making pooled BAC libraries from genomic DNA without individual colony picking and maintenance would costs approximately US$5,000, and preparing DNA from each pool would cost roughly US$20. Therefore, to implement this BAC-based approach routinely, the total cost involved in BAC library construction and preparing DNA from 24 BAC pools would be roughly US$5,480.

**Figure 2:**
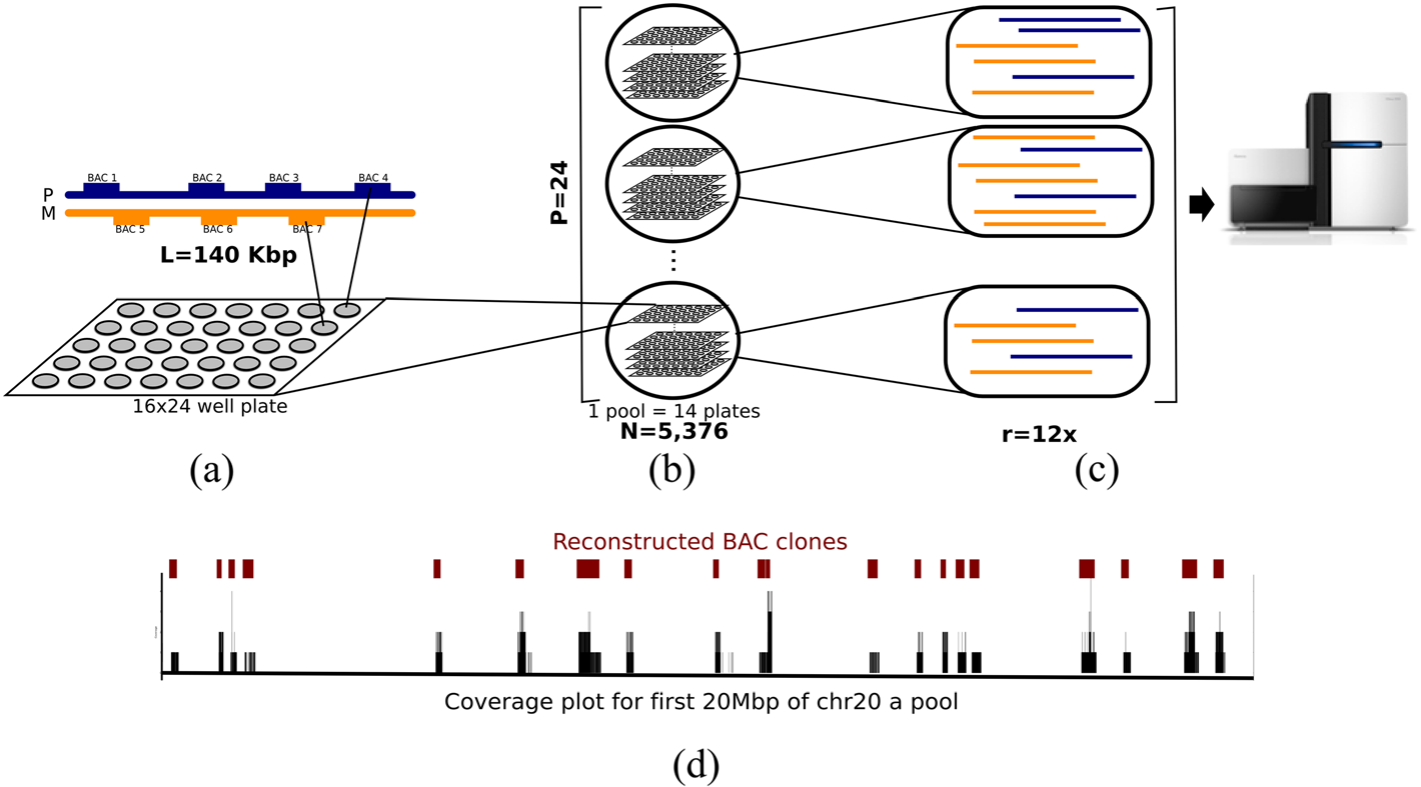
**Haplotyping with bacterial artificial chromosome (BAC) clones. (a)** Constructing the BAC library. DNA was extracted from PGP1 (NA20431, Personal Genome Project) and BAC clone libraries with clone length (*L*= 140 kbp. **(b)** Forming pools of BAC clones. The number of pools (*p*) formed was 24, with each pool consisting of fourteen 16 × 24-well plates, so that there was a total of *n* = 5,376 clones per pool. **(c)** Sequencing and mapping each pool. Sequencing libraries were prepared for each pool with a read coverage of *r* = 6**x**. After sequencing, reads were mapped to hg19. **(d)** Reconstructing BAC clones. Clones were reconstructed from the mapped reads of each pool using coverage-based techniques (clones detected in region of chromosome 20).

Following this design (Figure 2), we constructed 24 sequencing libraries from the 24 pools, which collectively contained 129,024 BAC clones. A total of 2 billion pair-end 100 bp reads were generated for these libraries, with an average of approximately 74 million reads for each pool. Of these, roughly 47 million reads (63.5%) were uniquely aligned to the genome, giving an effective read coverage (*r*) of 6x per pool.

### Reconstruction of BAC clones

In each pool, the boundaries of BAC contigs were determined by detecting regions of enriched read coverage after the reads of the pool had been mapped to the genome [11] (see Methods). If clones in a pool do not overlap, a BAC contig will contain only one BAC clone, and the boundary of a BAC contig will be the boundary of a BAC clone. However, given that *c*_*p*_ = 0.25 in our experiment, we estimated that the percentage of BAC contigs containing more than one clone would be *P*_*0*_ = 39.34%. When clones overlap, it is not possible to assume that the consensus sequence of the BAC contigs provides haplotype information.

With the goal of maintaining the haploid nature of each pool, we developed a computational approach to detect and remove regions covered by more than one clone (see Methods). Previous clone-based methods detected and removed overlaps by finding heterozygous variants and either removing the whole contig [11] or breaking the contigs at those locations [12]. These methods were sufficient for previous methods because *P*_*0*_ was relatively low (Table 1). However, we developed a more sophisticated method to detect and remove only the overlapping regions of a contig. Briefly, our method first detects the boundaries of overlapping regions by searching for bulges in coverage. Using these boundaries, we removed regions of the contigs that contain a significant fraction of heterozygous variants, as these probably represent regions of overlapping clones.

Before removing the overlap regions, we detected a total of 92,937 BAC contigs with an average length of 161,397 bp (N50 = 199,744 bp). This is consistent with estimates derived from Lander and Waterman statistics for the expected number and length of contigs (100,396 contigs and 159,127 bp). After removing the overlap regions, there were a total of 85,445 reconstructed clones with an average length of 140,777 bp (N50 = 161,300 bp). Note that some contigs had to be removed completely because the non-overlapping portions of the clones could not be recovered (see Additional file 1: Figure S5 for the distribution of lengths for the final reconstructed BAC clones).

### Variant detection

To recover variants, we pooled together all the sequence data from the 24 pools and called variants using BWA/GATK software [19] (see Methods). A crucial part of phasing is variant calling, and more specifically, differentiating heterozygous and homozygous variants. However, our method could call heterozygous variants only where both haploids were covered by BAC clones. For instance, if only the haplotype with the reference allele of the variant was covered, the variant would not be called. Likewise, if only the haplotype with the non-reference allele of the variant was covered, the variant caller would not be able to confirm its zygosity, and it would be called homozygous by default and be discarded for phasing. Of the 2,906,810 variants recovered, 1,287,220 variants were called as heterozygous and 1,619,590 were called as (non-reference) homozygous.

To overcome the challenges caused by low clone coverage, we augmented the recovered variants by using existing Complete Genomics Institute (CGI) WGS data of PGP1 [13,20]. A total of 3,283,326 variants were called by CGI [21]. Of these variants, 2,086,302 were heterozygous and 1,196,934 were homozygous. A total of 3,208,817 variants were recovered by augmenting the variants detected by the pooled BAC data with those detected by CGI WGS data, and of these, 1,942,116 were classified as heterozygous (see Methods) and used in the haplotype assembly. When compared with dbSNP135, 3,083,460 (4%) of the 3,208,817 variants were found to be novel. The percentage of novel variants, the number of variants, and the homozygous to heterozygous ratio were comparable with other individuals of European descent (Table 2; see Additional file 1: Table S1).

**Table 2:** Variant statistics for PGP1 compared with other individuals of European descent

	**Total variants**	**Hom/Het ratio**	**Novel variants, %**
PGP1 (BAC Pools)	2,906,810	1.26	1%
PGP1 (CGI WGS)	3,283,236	0.57	4.9%^a^
PGP1 (BAC + CGI WGS)	3,208,817	0.71	4%^a^

*Abbreviations:BAC* bacterial artificial chromosome, *CGI* Complete Genomics Institute, *Hom/Het* homozygous/heterozygous.

^a^Compared with dbSNP135.

### BAC haplotype assembly

Haplotype contigs were assembled by chaining together heterozygous variants that were connected by a BAC clone; the more overlapping clones (that is, higher clone coverage) present, the longer would be the expected haplotype length. Given the number of reconstructed BAC clones (*n*′ = 85,445), and their average length (*L′* = 140,777), the effective clone coverage, c'=L'n'pG, was 4x. Previous methods report clone coverage of 6.6x [11], 12.56x [12], and 38-56x [13]. Although the clone coverage for this BAC haplotyping experiment was lower, we achieved longer haplotypes because of the longer length of the BAC clones. In total, 2,379 haplotype contigs were assembled to form haplotypes with an N50 length of 2,640,036 bp. The chromosome level breakdown of the number of contigs and N50 lengths is shown (Table 3), and the distribution of the haplotype lengths is provided (see Additional file 1: Figure S6). The longest haplotype contig spanned over 14 Mbp.

**Table 3:** Chromosome level breakdown of haplotype statistics

**Chrom**	**Number of clones**	**Number of heterozygous. variants**	**Fraction of heterozygous variants phased**	**Number of contigs**	**N50 Haplotype length**
		**(BAC + CGI WGS)**			
1	6,892	148,691	0.973	219	2,166,488
2	7,632	156,474	0.978	195	2,905,522
3	6,677	147,498	0.985	146	3,015,997
4	6,937	159,704	0.982	106	4,113,256
5	6,101	127,746	0.983	117	2,700,783
6	5,818	142,863	0.981	119	3,005,140
7	4,781	113,746	0.974	150	2,621,008
8	4,589	98,664	0.982	114	2,413,181
9	3,485	80,480	0.966	125	2,276,528
10	4,104	99,111	0.969	109	2,264,959
11	4,233	90,557	0.979	113	3,368,062
12	4,150	93,795	0.979	116	2,641,808
13	3,395	75,463	0.984	51	2,945,506
14	2,846	62,844	0.967	69	3,059,729
15	2,520	55,794	0.967	71	2,067,670
16	2,031	59,080	0.956	123	1,252,516
17	1,965	47,238	0.96	116	1,485,600
18	2,547	56,853	0.981	43	3,345,667
19	1,155	32,849	0.956	110	660,242
20	1,649	39,551	0.958	66	1,608,505
21	1,170	34,095	0.958	36	3,226,907
22	768	19,020	0.956	65	1,057,117
Total	85,445	1,942,116	0.975	2,379	2,640,036

*Abbreviations:BAC,* bacterial artificial chromosome; *Chrom,* chromosome; *CGI,* Complete Genomics Institute; *WGS,* whole genome sequencing.

### Accuracy

We used the minimum edit score (MES) to measure the accuracy between two independently derived haplotypes. The MES takes into account the two common error modes for haplotype assembly- mismatches and switch errors. When comparing two haplotypes, an error can be classified as either a mismatch or a switch. Given the cost of a mismatch (*c*_*m*_), the cost of a switch (*c*_*s*_), the number of mismatches (*m*), and the number of switches (*s*) the total cost is given by

(5) mcm+scs#var

Under the MES criterion, the objective is to classify each error as a mismatch or a switch, such that the total cost is minimized. For example, if there are 10 consecutive errors, these can either be classified as 10 mismatches or 1 switch. If *c*_*s*_ < 10*c_*m*_, then under the MES objective, these errors would be classified as one switch. The classification of errors as mismatches or switches will depend on the cost. In the calculations of this paper, we used *c*_*m*_ =1 and *c*_*s*_ = 1.

**Figure 3:**
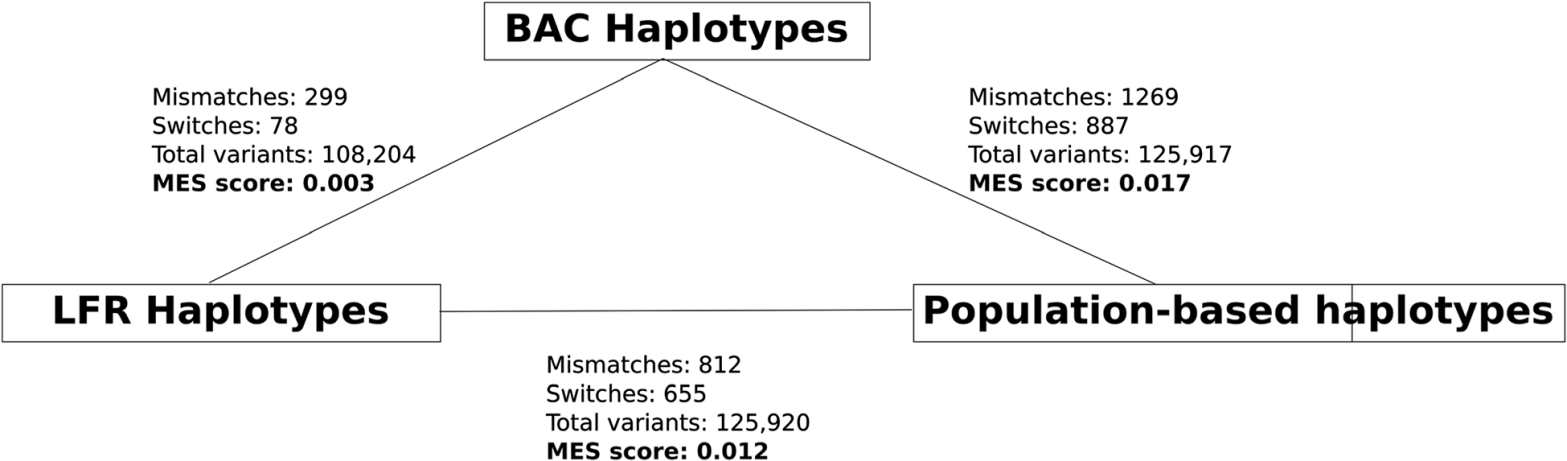
**Accuracy comparison between bacterial artificial chromosome (BAC),, long fragment read (LFR),, and population-based haplotypes.** The MES score is given by the classification of errors as mismatches or switches such that mcm+scs#var is minimized, where *c*_*m*_ = *c*_*s*_ = 1.

We tested the accuracy of our haplotypes (henceforth referred to as BAC haplotypes) by comparing them with the haplotypes of PGP1 constructed using the LFR clone-based method (LFR haplotypes) and a population-based method. The population-based haplotypes were computed with BEAGLE [2] using CGI WGS genotype data of PGP1 and population data from the 1000 genomes project [22]. In chromosome 1, the MES between the BAC and LFR haplotypes was relatively lower (0.003) than the MESs involving population-based haplotypes (LFR = 0.012, BAC = 0.017) (Figure 3). The small discrepancy between two haplotypes could be an error in either the LFR or in the BAC haplotypes. Specifically, haplotype errors are caused by the improper linking of heterozygous variants or errors in variant calling, both of which can happen if there are not enough clones spanning a particular site.

**Figure 4:**
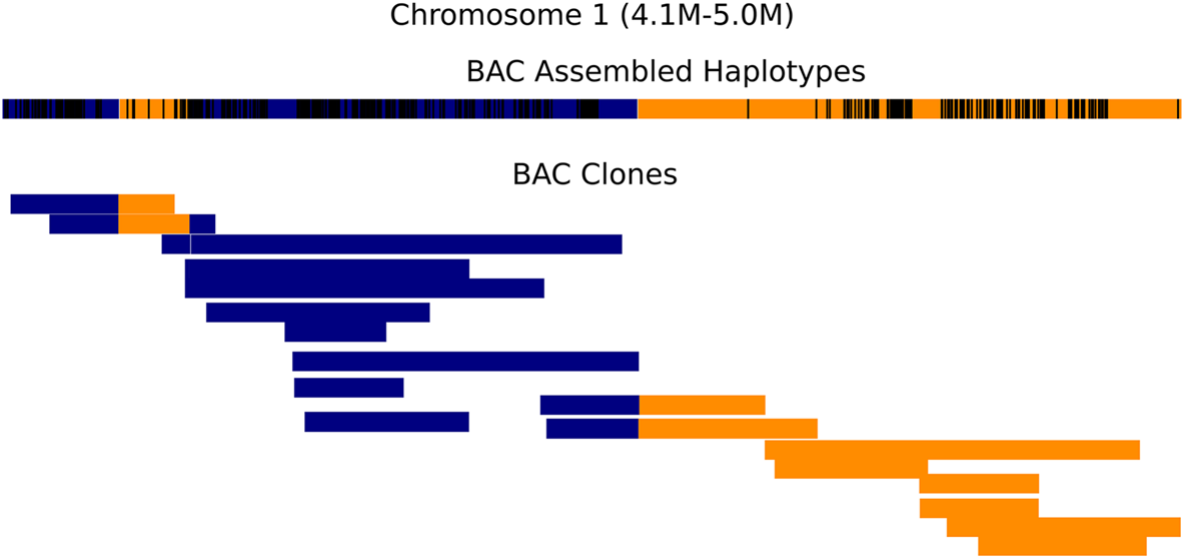
**Consistency between BAC-assembled haplotypes and BAC clones.** A snapshot of a 1 Mbp region on chromosome 1 illustrating three switch errors between BAC-assembled haplotypes and the population-based haplotypes (indicated by three color changes along the haplotype). At all three switches, 100% of the BAC clones that span this switch are consistent with the BAC-assembled haplotypes. The heterozygous variants that are phased are represented as black vertical lines in the BAC-assembled haplotypes.

We computed the clone coverage at discrepant sites and found that 95% of the discrepant sites were covered by three or more clones, and there was no correlation between discrepancy and clone coverage (see Additional file 1: Figure S7). Furthermore, we compared the accuracy between the BAC haplotypes and BAC clones. Of the 358,697 overlapping variants, there were 1,486 mismatches and 353 switches, giving an MES of 0.005. The small percentage of mismatches (0.41%) and switches (<0.1%) can be attributed to sequencing error and errors in clone reconstruction, respectively. Owing to the clone coverage of 6x, most of the errors are recovered during haplotype assembly (Figure 4). Our results therefore suggest high accuracy for the computed BAC haplotypes.

### Haplotyping the HLA region

The human leukocyte antigen (HLA) region is a 5 Mbp region on chromosome 6 that contains many genes that have important regulatory roles in the immune system. Haplotype information of the HLA regions is medically relevant because the specific combination of certain alleles is known to be linked with several autoimmune and other diseases. Owing to the repetitive nature of the HLA region, the haplotypes here are difficult to obtain with current next-generation sequencing technology. However, the length of BAC clones can be used to span over these repetitive regions and connect many more genes, achieving long, accurate haplotypes.

**Figure 5:**
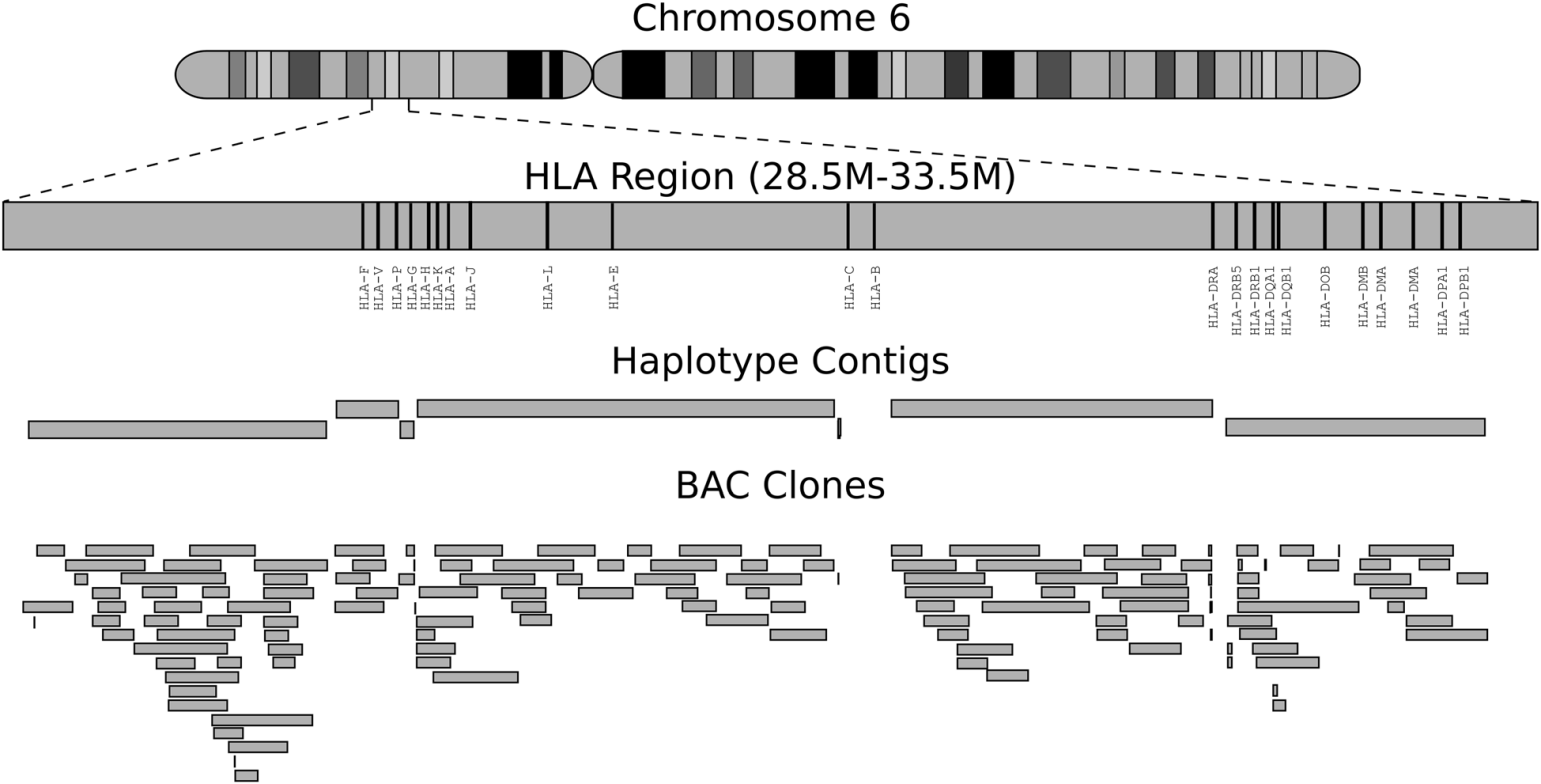
Haplotypes of the human leukocyte antigen (HLA) region.

In our experiment, the 5 Mbp HLA region was covered by 145 BAC clones, which assembled into 7 haplotype contigs. Similar to a previous study [14]. More than 90% of the entire HLA region was spanned by six contigs, and the longest haplotype contig in this region spanned 1.37 Mbp (N50: 1.1 Mb). Figure 5 shows the BAC clone coverage of this region. Of 23 HLA genes, 20 were spanned by BAC clones; 18 of these were phased completely (>90% of variants phased in 1 haplotype block), and 2 partially. In addition, 96.7% (11,861 of 12,272) of the heterozygous variants in this region were phased (see Additional file 1: Table S2).

## Discussion

In our parameterized analysis of clone-based haplotyping methods, the current bottleneck for achieving long haplotypes was the clone lengths (*L*). Because of the distribution of variants, adjacent variants that are longer than *L* can never be spanned, and thus the haplotype lengths saturate when all variants within a distance greater than or equal to *L* from each other are connected.

The importance of *L* is illustrated in Figure 1a, which shows a power law relationship between haplotype length and *L*. Furthermore, it illustrates the current gap between *in vitro* technologies for isolating DNA and clone-based methods. As shown, when *L* = 10 kb (current limit on reported length of Illumina’s Moleculo technology [15]), the maximum achievable haplotype length is 188 kb. Meanwhile, clone technologies have the potential to achieve significantly longer haplotypes with N50 lengths of 1.12 Mb (*L* = 40 kb, Fosmid clones) to 22.8 Mb (*L* = 140 kb, BAC clones). The importance of *L* is not just limited to clone-based and dilution-based methods; haplotyping using sequence reads can be modeled using our framework by setting *L* as the read length, *n* = 1, and *p* as the number of reads sequenced. For example, long reads were used to assemble haplotypes was on the HuRef genome [23]. The HuRef genome used a more complex paired-end Sanger sequencing (7.5x coverage) protocol with mixed insert sizes (*L*) and achieved haplotype lengths of N50 = 350 kb. More recent methods [24] use a single molecule approach to achieve long reads. The importance of *L* is further illustrated in Figure 1b, as other clone-based methods are well into the saturation levels of their corresponding expected contig-length curve. We concluded that it was more effective to increase *L* and use a moderate *p*. In our experiment (*L* = 140 kb, *p* = 24), we achieved haplotypes that had comparable accuracy to leading clone-based methods, and were more than twice as long, with an N50 length of 2.6 Mbp and the longest haplotype spanning over 14 Mbp. By contrast, the LFR haplotypes, derived from shorter clones (*L* (N50) = 60 kb) and more pools (*p* = 384), had an N50 length of 411 kb for the same individual [13].

By reducing *p*, the total cost of sequencing and clone library construction was reduced, but clone coverage was also decreased. Although we were able to compensate for the small clone coverage in terms of haplotype length by using larger *L*, our lower coverage recovered fewer variants compared with WGS experiments from other individuals of European descent (see Additional file 1: Table S1). This low clone coverage also decreased the probability of recovering a heterozygous variant (*P*_*v*_) and may explain the higher homozygous to heterozygous ratio for BAC pool data (see Additional file 1: Table S1). However, the variants could be augmented by acquiring WGS data from the same individual.

The final parameter affecting haplotype resolution and thus length is *f*, the fraction of variants recovered per clone, which is affected by many other factors such as read coverage per pool (*r*), amplification bias, and the filtering protocol for reads and variants. The discrepancy between simulated and actual haplotypes lengths in Figure 1b may be due to different values of *f* (see Additional file 1: Figures S3 and S4). For example, it is not surprising that the most discrepant results are from the LFR experiment, which used low values of sequencing coverage (*r* < 2x, in contrast with our protocol where *r* = 6x), causing a smaller value of *f*, which in turn decreases haplotype lengths.

To test accuracy, we performed a three-way comparison between the BAC, LFR, and population-based haplotypes (Figure 3). The high concordance between the BAC and LFR haplotypes suggests that both methods have similar accuracy. The higher MES between the clone-based and population-based haplotypes could be due to a variety of factors, including limited population sample size and limited burn-in iterations run by the algorithm due to limited computational resources. Furthermore, population-based haplotypes have difficulty phasing rare, individual-based, and somatic variants. Upon further examination of the population-based haplotypes, we found that the positions of the switch errors correlated with positions where the BEAGLE algorithm had difficulties deciding which phase assignment to choose. The biological implications of these regions have not yet been studied and could possibly represent undiscovered recombination hot spots, or simply areas where the population data are weak. In summary, clone-based haplotypes can be used to provide accurate, megabase-long haplotypes.

## Conclusions

Through the integration of statistical modeling and experimental validation, we found that long-range connectivity encoded in large clones or DNA fragments is crucial for constructing long haplotypes. We also provide a practical guideline on the parameter choices and expected haplotype sizes for further design and development of haplotyping methods.

## Methods

### BAC library construction and pooling strategy

BAC libraries were constructed with an average length of approximately 140 kb from genomic DNA of the PGP1 sample by Amplicon Express (Pullman, WA, USA). BAC clones were grown in separate wells on 16 × 24-well plates. The 384 clones on a plate were then combined to form a mini-pool via a two-dimensional pooling strategy, as described by Oeveren *et al*. [25]. Briefly, the strategy combines clones on a plate by rows and columns using a liquid handling robot (Biomek 2000; Beckman Coulter, Brea, CA, USA). Super-pools were formed by further combining clones from 14 mini-pools so that each super-pool contained a total of 5,376 BAC clones. On average, 150 to 250 ng of high-quality DNA was purified in each super-pool by applying DNA isolation via a modified alkaline lysis DNA extraction protocol [26]. In total, 24 super-pools were constructed.

### Construction of sequencing library and variant calling

DNA derived from an individual super-pool was precipitated with ethanol and dissolved in water, then used (10 ng) for random fragmentation by Tn5 transposon based fragmentation method (Epicenter, Madison, WI, USA). Fragmented DNA was purified by Ampure XP beads (Beckman Coulter) and attached with illumina adaptors by PCR amplification to construct sequencing libraries. Barcoded libraries were pooled for sequencing using a HiSeq 2000 instrument (Illumina).

Sequencing libraries were constructed for 24 pools. The resulting sequencing data were processed for variant calling using an established pipeline based on BWA/GATK, following the GATK best practices instructions (version 3). All raw sequencing data have been deposited to NCBI Sequence Read Archive under the project number SRP029150.

The goal for variant calling is to recover all the heterozygous variants for phasing. In particular, a heterozygous variant can fall into one of four categories: 1) both alleles are sampled by clones, 2) only the non-reference allele is sampled by clones, 3) only the reference allele is sampled by clones, and 4) none of the alleles are sampled. We focused on determining the heterozygous variants that fell into the first three categories, as no clones covered those the fourth category and their phase was non-determinable with the sampled BAC clones. In the previous paragraph, we described how heterozygous variants from category 1) are recovered. To recover variants from the second and third category, we used CGI WGS data of PGP1 [13]. The CGI WGS reads were mapped to hg19/b37 reference genome for variant calling using the CGI proprietary algorithm. For the heterozygous CGI variants that were not recovered using BAC pool data, we needed to verify that at least one clone covered the variant For instance, if a CGI heterozygous variant is called homozygous in using pooled BAC reads, it falls into category 2) and we can phase it. If a CGI heterozygous variant is not called using pooled BAC reads but is covered by at least eight reads from the pooled BAC data, we consider it a heterozygous variant from category 3) and recover it.

### Reconstructing BAC clones from sequencing reads

After mapping the sequence reads in a pool to the reference genome, we identified regions of enriched coverage (that is, BAC contigs) by using targetcut in the SAMtools library [27]. targetcut identifies regions of enriched coverage by calculating read depth for 1 kbp windows and then looking for consecutive regions where two-thirds of the windows have a read depth above the predicted background level (95th percentile of read depths, if reads were distributed uniformly across the genome). The regions are then appropriately trimmed to find the first and final base pair read in each region.

To recover the non-overlapping portions of a BAC clones, we looked for significant changes in coverage using a method similar to those for detecting changes in copy number variation [28]. This algorithm (see Additional file 1: Figure S8) begins by obtaining the read count for non-overlapping windows of 100 bp within the boundaries of a BAC contig. It is assumed that the read count of a window with overlapping clones has low variance. Therefore, when there is a significant increase in the read count, this indicates that more than one clone is covering the area. Let *R(x)* be the resulting window versus read count function, where *x* is the window number (in genomic position order), then, to detect significant changes in read count, we convolute *R(x)* with the derivative Gaussian function,

$$G\left(x\right)=-\frac{x}{\sqrt{2\pi }}e^{x^{2}/2}.$$

The breakpoints of the BAC contig are indicated by positions where the convoluted function reaches above or below a certain threshold (|*t*| = 30). After breaking up the contigs at the breakpoints, the resulting regions are either non-overlapping potions of a BAC clone or the overlapping portion.

### Assembling haplotypes from BAC clones

We used a generalized version of HapCUT [29] to assemble BAC clones into haplotypes. Following the notation of Bansal and Bafna [29], the input to HapCUT can be represented as a matrix, *X*, where each row represents a BAC clone and each column represents a heterozygous variant. It is assumed that all heterozygous sites are bi-allelic, as there are only two haplotypes, and thus the alleles are arbitrarily relabeled as 0 and 1. An entry in the matrix, *X*[*i*][*j*], is either '0’, '1’ or '-’ depending on the allelic value of position *j* in BAC clone *i*. The goal is to partition the rows (clones) of the matrix into two disjoint sets corresponding to the two haplotypes. If the fragments are error-free, the columns of each set are homozygous. However, sequencing errors, for instance, can produce errors in the fragments, and perfect bi-partitions cannot be achieved. Therefore, the goal is to partition the clones such that error correction is minimized- this is also known as the minimum error correction (MEC) objective.

The generalized version of HapCUT takes as input the BAC clones represented in the matrix form described previously. The algorithm starts by assigning a random haplotype configuration and iteratively improves it by finding positive cuts in the graph representation of the matrix and current haplotype configuration. In the graphical representation, the nodes are the variants and there is an edge between two variants if at least one clone covers both variants. The weight of edge (*i*, *j*) is the number of clones that are inconsistent with the current phase of *i* and *j*, subtracted by the number of reads that are consistent with the current phase of *i* and *j* scaled by some factor. If switching the phase for variants on one side of the cut will improve the MEC score, the haplotype configuration is updated. The algorithm iteratively finds positive cuts and updates the haplotype configuration until the MEC score does not improve.

## Abbreviations

BAC: Bacterial artificial chromosome; CGI: Complete Genomics Institute; HLA: Human leukocyte antigen; L: Clone length; LFR: Long fragment read; MEC: Minimum error correction; MES: Minimum edit score; n: Number of clones per pool; p: Number of pools; PGP: Personal Genome Project; WGS: Whole genome sequencing.

## Competing interests

The authors declare that they have no competing interests.

## Authors’ contributions

VB and KZ designed the project. RL, JL, and CL performed experiments. CL developed computational tools and perform data analysis with assistance from KR, SB, and JA. CL, RL, VB, and KZ wrote the manuscripts with critical input from JA and GC. All authors read and approved the final manuscript.

## Supplementary Material

Additional file 1 Supplementary figures, tables and methods. Click here for file
