# Supplementary material for: On the design of clone-based haplotyping
Source: Genome Biol. 2013 Sep 12;14(9):R100. doi: 10.1186/gb-2013-14-9-r100 (PMC4053695; doi:10.1186/gb-2013-14-9-r100)
Supplement: Additional file 1 — Supplementary figures, tables and methods. [file gb-2013-14-9-r100-S1.pdf]

SUPPLEMENTARY INFORMATION

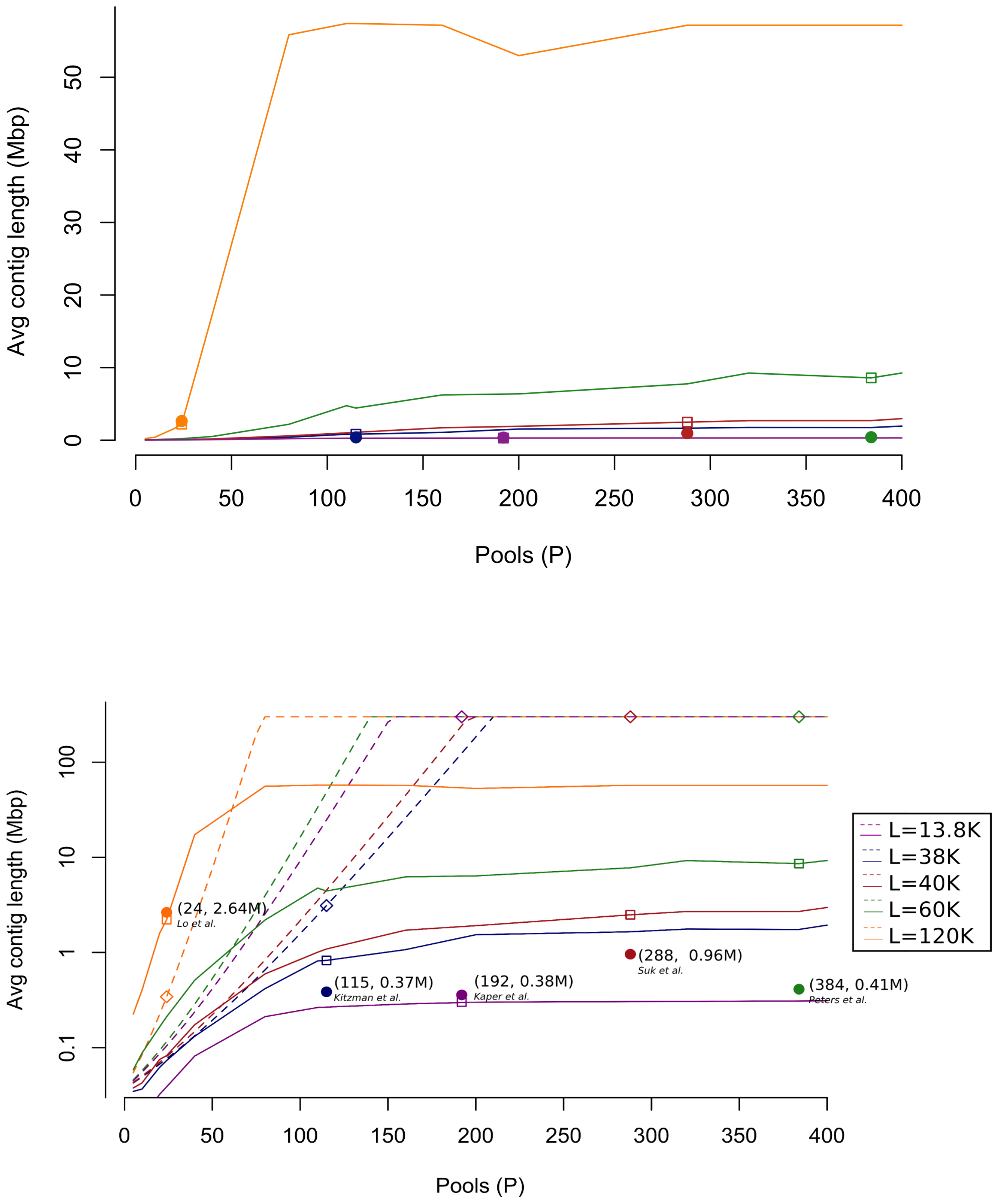

Figure 1 (a) Average contig length vs number of pools given read length. This figure shows Figure 1 with the log scale removed. (b)
The dotted lines show the Lander and Waterman estimates for haplotype length capped at 300 Mbp, the length of chromosome 1. The solid curves show the simulated contig

lengths given the actual distribution of heterozygous variants on chromosome 1 obtained from CGI whole genome sequencing data of PGP1. In contrast, the diamonds and squares represent the Lander Waterman estimate and simulated estimate respectively.
(b)

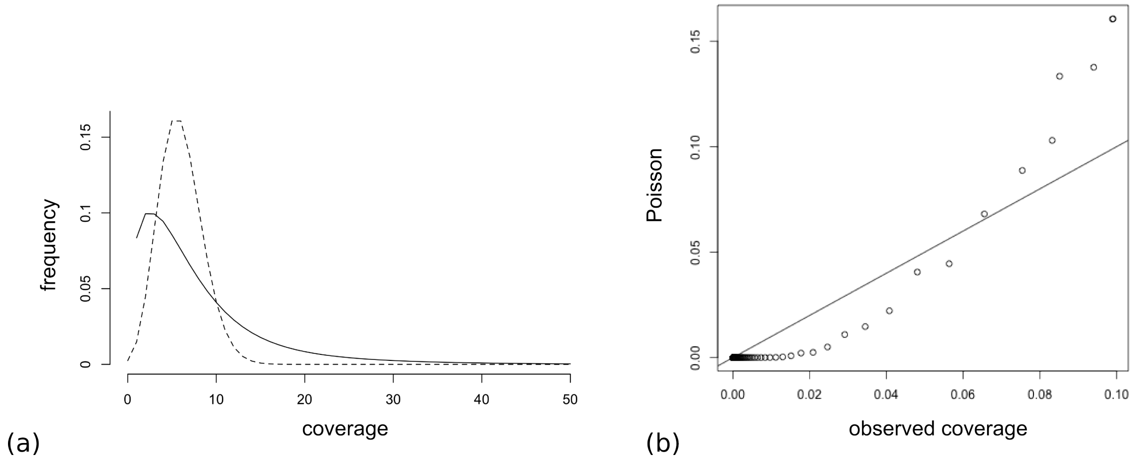

Figure 2 Effect of amplification bias on sequencing coverage (a) Actual and ideal distribution of the sequencing coverage. The solid line shows the actual distribution and the dashed line shows the ideal Poisson distribution. Using a naïve 4-read coverage rule for variant calling, under the idealized settings (no bias), we expect to see a variant recovery rate of 84%. However, under the observed bias on sequencing coverage, the 4-read coverage rule would yield a variant recovery rate of 61%. Indeed, using the GATK filter protocol for calling variants, the observed *f* is 65%. (b) Q-Q plot of the actual read depth distribution and the idealized Poisson distribution. Comparing the trend of the points to the *y*=*x* line illustrate the difference between the two distributions.

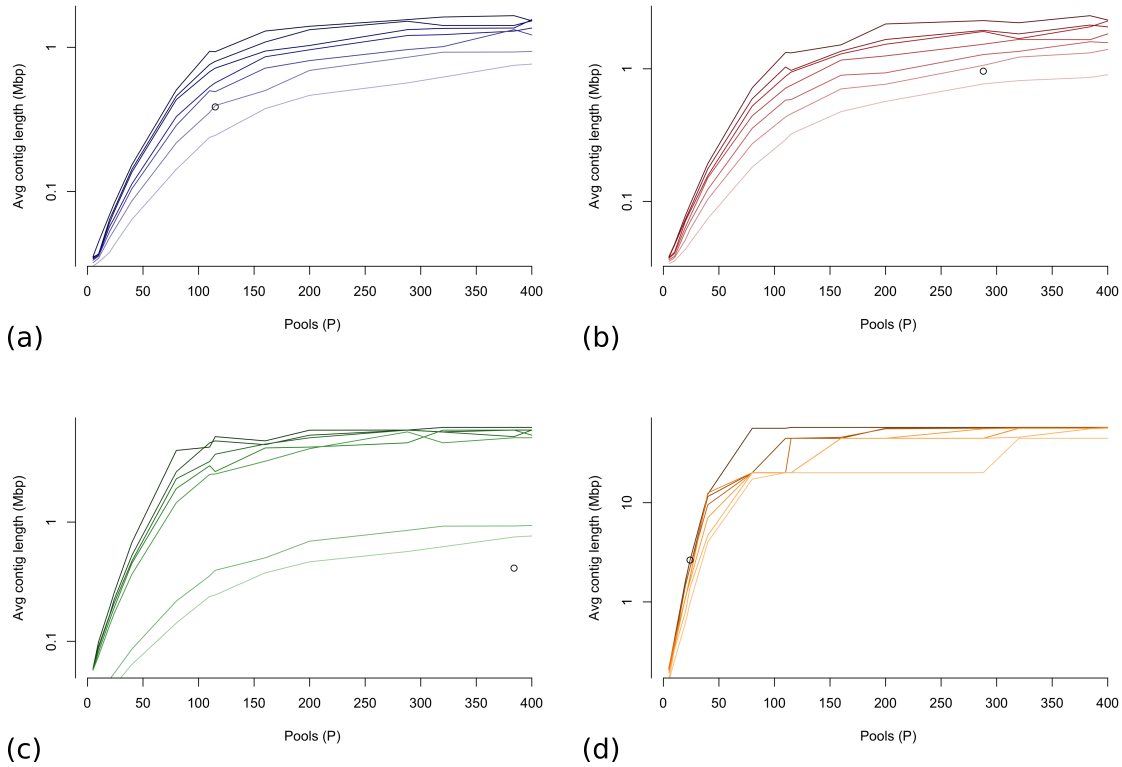

Figure 3 Simulation results that show how haplotype ***length*** is affected by *f*, the fraction of variants recovered per clone. In each subfigure, *L* is set to (a) 37 Kb, (b) 40 Kb, (c) 60 Kb, or (d)140 Kb while *N* is fixed at 5000 and *P* and *f* are varied. Each curve in a subfigure represents simulations under a different value of *f (*1.0, 0.75, 0.65, 0.55, 0.45, 0.35, or 0.25); the darker color indicates higher *f* value. The circle dots represent the actual reported N50 length for (a) Kitzman et al., (b) Suk et al., (c) Peters et al., and (d) our BAC clone haplotypes.

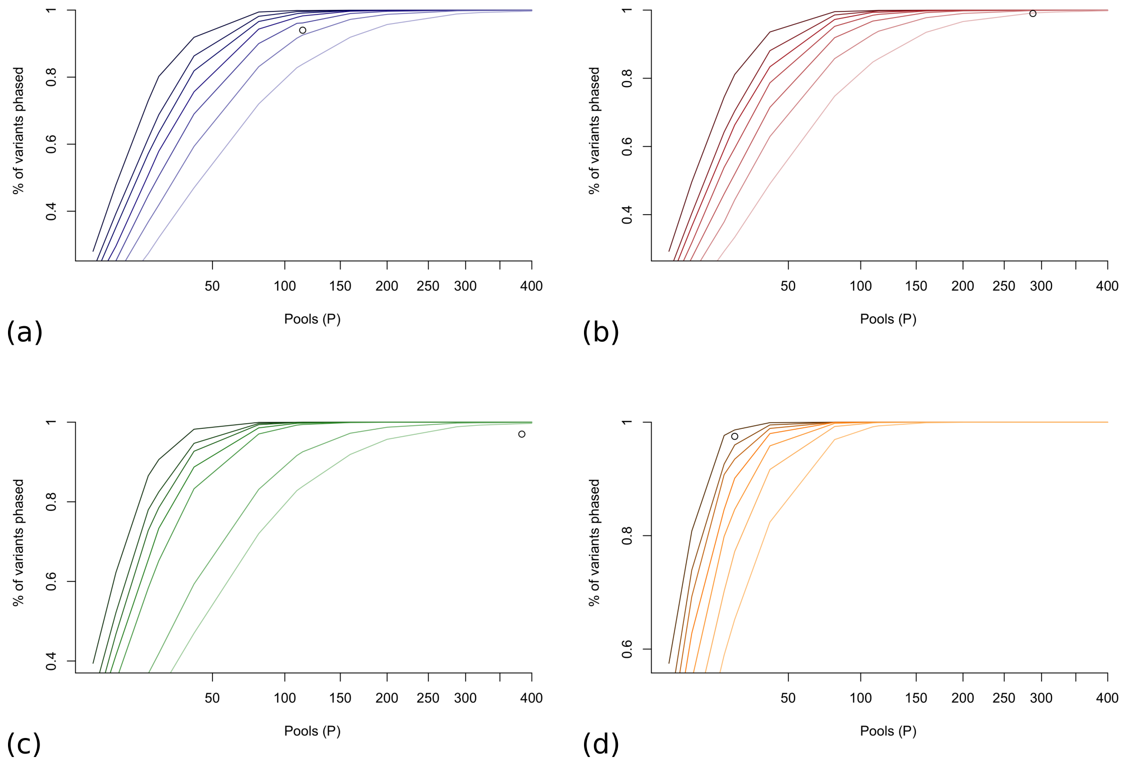

Figure 4 Simulation results that show how haplotype ***resolution*** is affected by *f*, the fraction of variants recovered per clone. In each subfigure, *L* is set to (a) 37 Kb, (b) 40 Kb, (c) 60 Kb, or (d)140 Kb while *N* is fixed at 5000 and *P* and *f* are varied. Each curve in a subfigure represents simulations under a different value of *f (*1.0, 0.75, 0.65, 0.55, 0.45, 0.35, or 0.25); the darker color indicates higher *f* value. The circle dots represent the reported haplotype resolution for (a) Kitzman et al., (b) Suk et al., (c) Peters et al., and (d) our BAC clone haplotypes. The actual haplotype resolution of our BAC haplotypes (97.5%) is slightly higher than the simulated haplotype resolution at f=0.65 (93.5%). The value of f is not reported in the other studies.

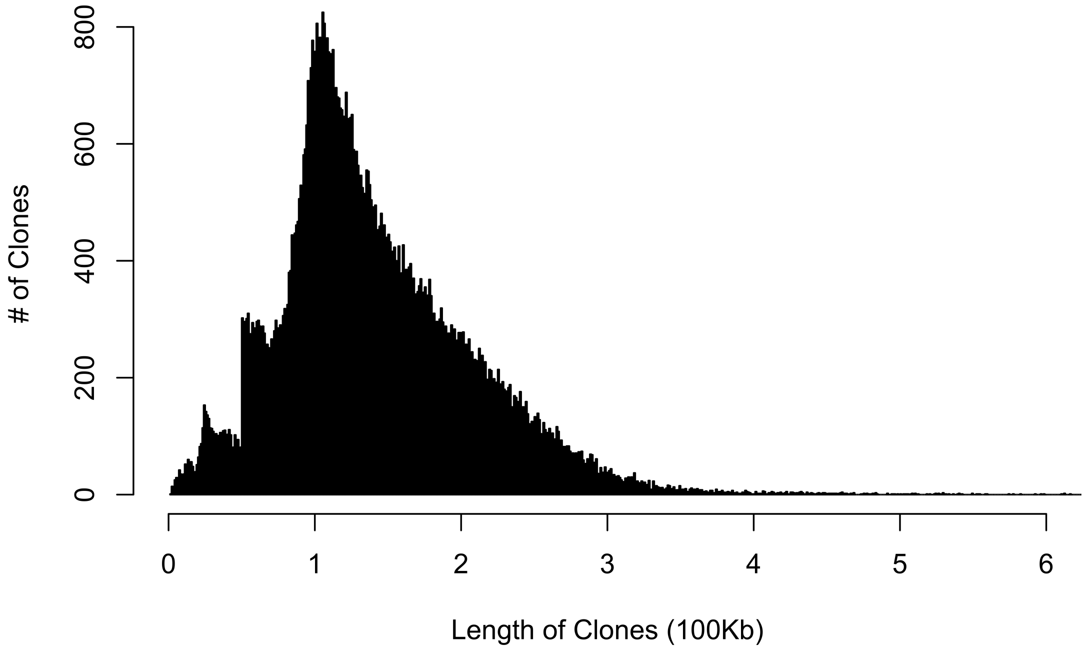

Figure 5. Distribution of reconstructed clone lengths.

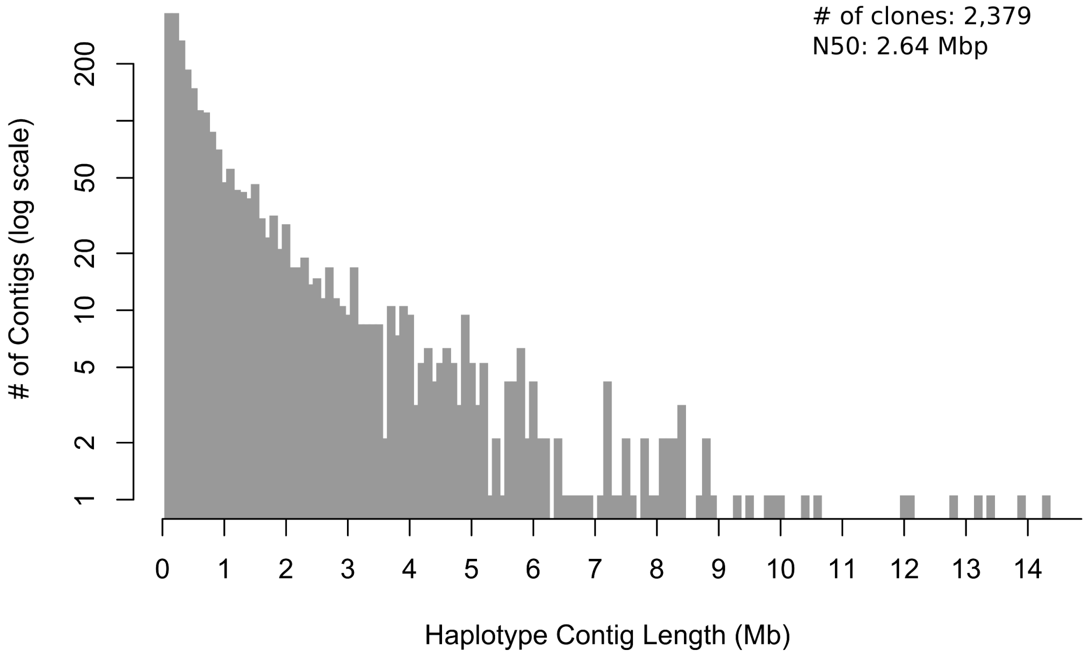

Figure 6. Distribution of the haplotype lengths.

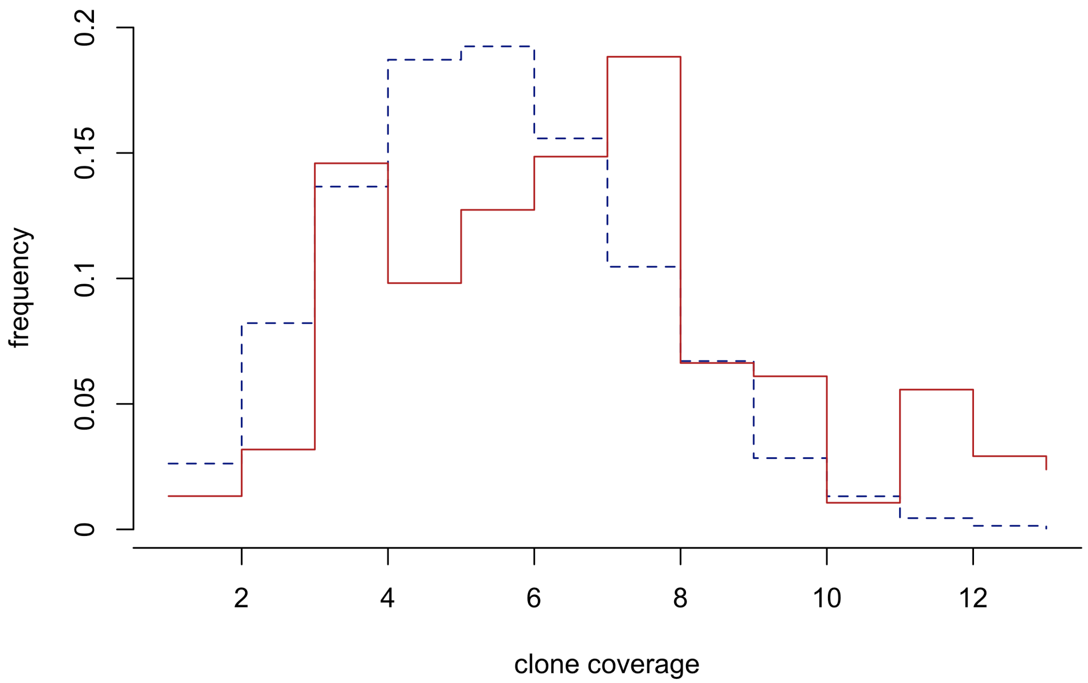

Figure 7. Distribution of clone coverage at discrepant locations (red, solid) and match locations (blue, dashed) when comparing BAC haplotypes with LFR haplotypes. The average clone coverage at discrepant locations is 6.2 while the average clone coverage at match locations is 5.0. Furthermore, 95% of the discrepant locations are covered by three or more clones, indicating high confidence in our calls.

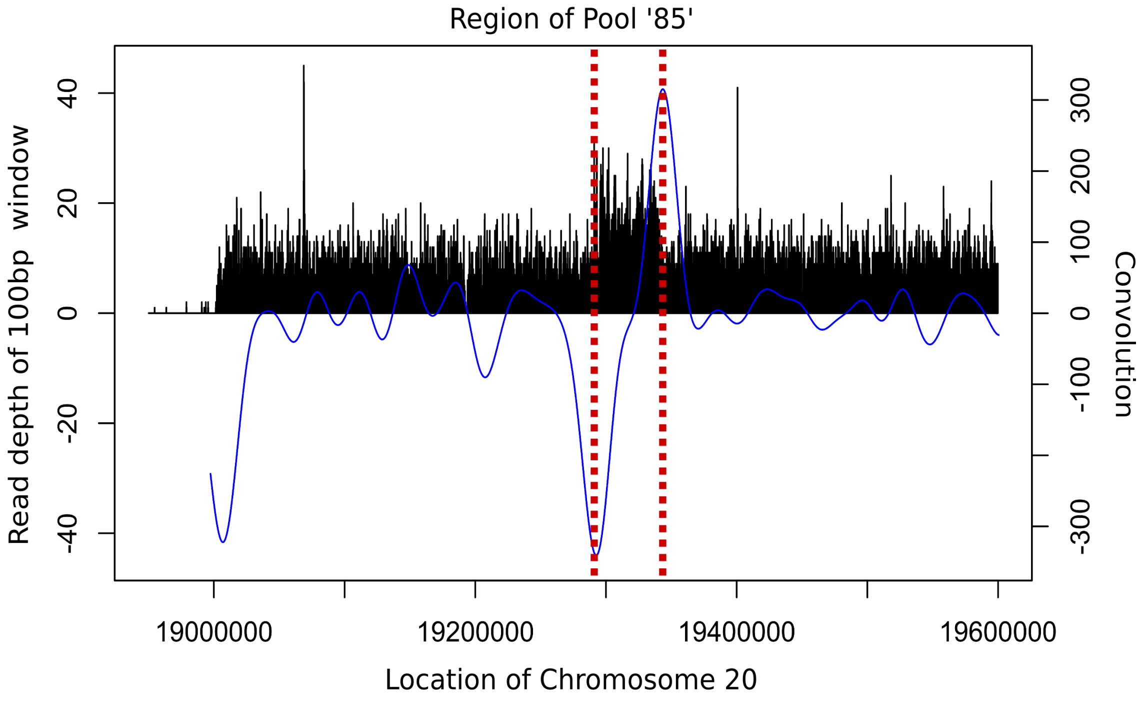

(a)

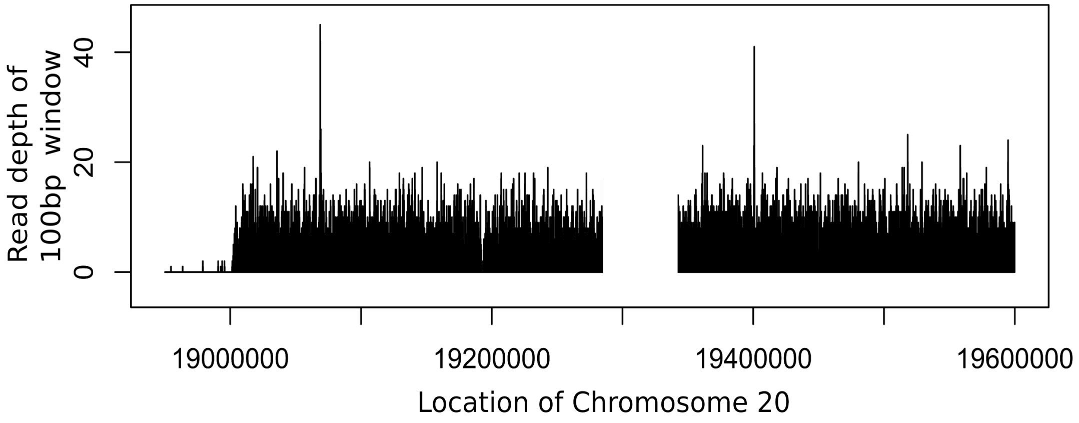

(b)

Figure 8. Illustration of clone reconstruction method. (a) Read depth of a region on chromosome 20 in pool '85'. The read depth is convoluted with the derivative Gaussian function to determine the boundaries (dotted red lines) of the overlapping region. (b) The two reconstructed BAC clones after removing the overlapping region.

Table 1. Variant statistics comparison with other individuals of European descent.

	Total Variants	Hom/Het Ratio	% Novel Variants
HuRef [Levy et al. 2007]	3,213,401	0.82	15%
JDW [Wheeler et al. 2008]	3,322,090	0.79	18%
NA07022 [Drmanac et al. 2010]	3,076,870	0.61	10%
20 Genomes [Pelak et al. 2010)]	3,473,639[1]	0.59[1]	13%[1,2]
MP1 [Suk et al.]	3,258,774	0.59	8% [2]

[1] Average
[2] compared to dbSNP129

Table 2. HLA statistics.

Gene	Total num. of clones	Number of haplotype blocks	% of bases covered by clones	Variants detected (Het. and Hom. variants)	Variants phased	% of variants phased
HLA Class I Genes						
HLA-A	7	1	100%	105	104	99%
HLA-B	0	0	-	-	-	-
HLA-C	0	0	-	-	-	-
HLA-E	2	1	100%	3	3	100%
HLA-F	5	1	100%	15	15	100%
HLA-G	1	1	100%	31	29	94%
HLA-H	4	1	100%	63	63	100%
HLA-J	6	1	100%	3	3	100%
HLA-K	5	1	100%	53	53	100%
HLA-L	5	1	100%	13	13	100%
HLA-P	1	1	100%	30	29	97%
HLA-V	1	1	100%	23	23	100%
HLA Class II Genes						
HLA-DRA	2	1	100%	53	53	100%
HLA-DRB1	7	1	100%	480	477	99%
HLA-DRB5	0	0	-	-	-	-
HLA-DPA1	5	1	100%	1	1	100%
HLA-DPB1	4	1	100%	156	154	99%
HLA-DQA1	8	1	100%	186	185	99%
HLA-DQB1	7	1	100%	123	121	98%
HLA-DMA	2	1	100%	14	5	36%
HLA-DMB	1	1	100%	28	5	18%
HLA-DOA	4	1	100%	14	14	100%
HLA-DOB	4	1	100%	15	15	100%

Methods: Simulation of haplotype lengths

In order to simulate haplotype length given clone length (L), number of pools (P), and number of clones per pool (N), the following approximations were made:

- We used the distribution of heterozygous variants of PGP1 determined by CGI whole genome sequencing (Peters et al.). Using the exact distribution of variants is essential in modeling overlapping clones that are useful for phasing PGP1 and ultimately in determining haplotype length.
- Modeling overlapping clones in a pool. Due to high clone coverage within a pool, the probability that a clone overlaps with another clone in the same pool (P_o) is greater than 0. While we use more sophisticated methods to deal with overlaps within a pool, for simulations we use a first order approximation and assume that overlapping clones within a pool are thrown out. Therefore, the effective number of clones in a pool is $N'=N(1-P_o)$
- We modeled the sequencing read coverage per pool (r) by only recovering a fraction of the variants spanned by a clone (f). We calculated the fraction of variants recovered in each clone for our BAC data and found that f=65%. We note that this fraction is probably higher in Kitzman et al. and Suk et al. because they have higher read coverage and thus a higher chance of recovering variants, and probably lower in Peters et al. as r is very low. Additional File 1: Figure 5 & 6 shows these designs using different fractions of recovered variants in a clone.

The simulator for haplotype length and resolution is available upon request.

The actual reported N50 is close to the simulated N50. But we note that discrepancies may arise due to the assumptions we made. For example, if the distribution of variant distributions is more/less sparse than PGP1's, the simulated haplotype lengths will differ from the actual haplotype lengths. Amplification bias, read coverage (r), filtering protocol for overlapping clones and variants, as well as natural noise in the data may cause discrepancy between simulated and actual haplotype lengths.

Derivation: Equation 2

The probability that a clone does not overlap with a given clone is $\left(1-\frac{2L}{G}\right)$. Thus, the probability that a clone overlaps with a given clone in the same pool is given by

$$
\begin{aligned}
P_o &= 1-\left(1-\frac{2L}{G}\right)^{NP} \\
&= 1-\left(1-\frac{2L}{G}\right)^{c_p\frac{2G}{2L}} \\
&\approx 1-e^{-2c_p}
\end{aligned}
$$

Derivation: Equation 3

Given the length of the diploid genome is G, the total length of the haploid genome is given by 2G. Let x be the probability that a particular position on the haploid genome is not covered by any clone.

$$
\begin{aligned}
x &= \left(1-\frac{L}{2G}\right)^{NP} \\
&= \left(1-\frac{L}{2G}\right)^{c\frac{2G}{2L}} \\
&\approx e^{-c/2}
\end{aligned}
$$

In order to recover a heterozygous variant, both copies of the variant must be covered by at least one clone each. Therefore, the probability that a heterozygous variant is given by

$$
\begin{aligned}
p_v &= 1-2x \\
&= 1-2e^{-c/2}
\end{aligned}
$$

References

Drmanac R et al. (2010) Human genome sequencing using unchained base reads on self-assembling DNA nanoarrays. *Science* 327(5961): 78-81.

Kitzman JO*, et al.* (2011) Haplotype-resolved genome sequencing of a Gujarati Indian individual. *Nat Biotechnol* 29(1):59-63.

Levy S et al. (2007) The diploid genome sequence of an individual human. *PLoS Biol* 5(10): e254.

Pelak K et al. (2010) The characterization of twenty sequenced human genomes. *PLoS Genet* 6(9).

Peters BA*, et al.* (2012) Accurate whole-genome sequencing and haplotyping from 10 to 20 human cells. *Nature* 487(7406):190-195.

Suk EK*, et al.* (2011) A comprehensively molecular haplotype-resolved genome of a European individual. *Genome Res* 21(10):1672-1685.

Wheeler DA et al. (2008) The complete genome of an individual by massively parallel DNA sequencing. *Nature* 452(7189): 872-876.
